# Prevalence of Frailty and Its Predictors Among Patients With Cancer at the Chemotherapy Stage: Systematic Review

**DOI:** 10.2196/69936

**Published:** 2025-07-24

**Authors:** Tingting Wang, Jinxia Jiang, Zihe Song, Xianliang Liu, Minhui Zhong, Chan Yu, Runa Zhang, Xia Duan

**Affiliations:** 1 Nursing Department, Shanghai Key Laboratory of Maternal Fetal Medicine, Shanghai Institute of Maternal-Fetal Medicine and Gynecologic Oncology, Shanghai First Maternity and Infant Hospital, 2699 West Gaoke Road, Pudong New District, Shanghai, 200092, China, 86 20261221; 2 School of Medicine, Tongji University, Shanghai, China; 3 Emergency Department, Shanghai Tenth People's Hospital, Shanghai, China; 4 School of Nursing and Health Studies, Hong Kong Metropolitan University, Hong Kong, China

**Keywords:** cancer, frailty, chemotherapy, influencing factor, systematic review

## Abstract

**Background:**

Chemotherapy causes physiological, psychological, and social impairments in patients with cancer. Frailty reduces the effectiveness of chemotherapy and increases the toxicity associated with radiotherapy and chemotherapy, the possibility of chemotherapy failure, and adverse outcomes. However, factors affecting chemotherapy-related frailty in patients with cancer remain unclarified.

**Objective:**

This systematic review aimed to identify risk factors driving frailty progression during chemotherapy in patients with cancer.

**Methods:**

A comprehensive systematic search was conducted on PubMed, Web of Science, Embase, China National Knowledge Infrastructure, China Science and Technology Journal Database (VIP), and SinoMed for observational studies (cohort, cross-sectional, or case-control) on factors affecting the debility-of-chemotherapy stage in patients with cancers between the inception of the database and February 2025, with an updated search executed in May 2025. Literature screening, quality evaluation using the Newcastle-Ottawa Scale and Agency for Healthcare Research and Quality checklist, and data extraction were conducted independently by 2 authors. Meta-analysis, effect size combination, sensitivity analysis, and publication bias analysis were performed using RevMan (version 5.4; The Cochrane Collaboration) and R (version 4.4.3; R Foundation).

**Results:**

The analysis comprised 14 studies (8 cross-sectional, 2 repeated cross-sectional, 3 cohort, and 1 mixed-design), including 3879 patients with cancer and 23 influencing factors. Methodological quality assessment using Agency for Healthcare Research and Quality (mean 8.8, SD 1.3, 95% CI 7.9‐9.7; SE 0.4) and Newcastle-Ottawa Scale (mean 8.0, SD 1.0, 95% CI 6.7‐9.3; SE 0.6) revealed 73% (8/11) of cross-sectional studies as high-quality. The meta-analysis showed a 35% (95% CI 22%‐50%) prevalence of frailty during chemotherapy in these patients. Cancer stage (odds ratio 1.99, 95% CI 1.64‐2.42), chemotherapy frequency (odds ratio 2.60, 95% CI 1.83‐3.70), transfer (odds ratio 2.18, 95% CI 1.50‐3.17), hemoglobin (odds ratio 0.29, 95% CI 0.18‐0.47), white blood cell (odds ratio 0.37, 95% CI 0.21‐0.65), comorbidity (odds ratio 1.93, 95% CI 1.30‐2.86), and hypoproteinemia (odds ratio 1.74, 95% CI 1.31‐2.30) were risk factors for frailty in patients at the chemotherapy stage.

**Conclusions:**

Frailty during chemotherapy was strongly associated with advanced cancer stage, frequent treatment cycles, metastasis, anemia, leukopenia, comorbidities, and hypoproteinemia. Clinically actionable findings emphasized hemoglobin and albumin monitoring as preventive targets, while heterogeneity in assessment tools and population bias limited generalizability. The integration of frailty screening into chemotherapy workflows is urgent to mitigate treatment-related functional decline.

## Introduction

Frailty is clinically defined as a dynamic, multidimensional syndrome characterized by a progressive decline in physiological reserves and increased vulnerability to adverse health outcomes [1,2]. The Fried phenotype criteria [3], the integral model of frailty [2], and the cumulative deficit model [4] are the common diagnostic tools in clinical practice. Frailty manifests through 3 core pathophysiological mechanisms: multisystem dysregulation (characterized by endocrine-immunological crosstalk disruption), homeostatic failure (evidenced by the loss of physiological complexity), and stress response deterioration (manifesting as impaired allostatic load compensation) [5,6]. In oncogeriatric practice, frailty manifests as a cancer-specific vulnerability state driven by tumor-host interactions and accelerated aging processes.

Frailty remains prevalent among patients with malignant tumors owing to prolonged and complex treatments such as surgery, radiotherapy, and chemotherapy. A meta-analysis incorporating 11 studies involving 2898 patients with cancer revealed a pooled prevalence of frailty of 34% [7]. The malignant tumor and its associated treatments are significant stressors that decrease physiological reserves. Frailty is a critical factor contributing to the heterogeneity of health outcomes and treatment responses in patients with malignant tumors [7]. The prevalence of frailty in patients undergoing chemotherapy ranges from 17.2% to 39.3% [8,9]. Various chemotherapy-related factors, such as chemotherapy frequency, hypoproteinemia, and specific chemotherapy regimens, influence the level of frailty during treatment [10,11].

Patients with malignant tumors undergoing chemotherapy usually experience functional impairments across physical, psychological, and social domains. The dynamic nature of frailty exacerbates the risk for adverse outcomes, making it a vital predictor of health status and treatment outcomes [7,12]. Frailty increases the toxicity of radiotherapy and chemotherapy, reduces treatment tolerance, impairs cognitive function, and diminishes the quality of life while shortening survival time [7,13]. Furthermore, it decreases the effectiveness of chemotherapy [10], increases the possibility of treatment failure following tumor debulking [9], and contributes to poor outcomes, severely impacting the quality of life of patients. Frailty is an independent predictor of mortality in patients undergoing cancer surgery and chemotherapy, significantly increasing death risk [7,9,12]. Consequently, identifying risk factors for frailty in patients with malignant tumors is essential to improving their quality of life and treatment outcomes.

Frailty is influenced by diverse factors [14]. These risk factors include nonmodifiable and modifiable elements. Nonmodifiable factors include genetics, aging, and sex, while modifiable factors encompass socioeconomic status, lifestyle, comorbid conditions, geriatric syndromes, malnutrition, medications, and psychological conditions [7]. Hou et al [15] developed a frailty risk prediction model for patients with lung cancer, identifying age, fatigue, depression, nutritional status, D-dimer levels, albumin levels, comorbidities, and disease duration as independent predictors of frailty. Similarly, Xingyao and Huijiao [16] found that age, tumor stage, the presence of 2 or more comorbidities, moderate dependence on daily activities, and depression were independent risk factors for preoperative frailty in patients with cancers. Xu et al [17] determined regular exercise and psychological resilience as protective factors and demonstrated that advanced age and depression increase frailty risk. Moreover, a meta-analysis revealed that factors such as age, education, comorbidities, BMI, albumin levels, nutritional status, symptom clusters, depression, and daily living abilities significantly influence frailty in oncology patients [7]. Given the variations across different populations and disease stages, it remains essential to identify precise factors influencing frailty during chemotherapy in patients with malignant tumors.

Most domestic and international studies on factors influencing frailty in patients with cancers are small-sample cross-sectional studies [7]. These limitations affect the reliability of their findings, yielding inconsistent and poorly generalizable conclusions. Therefore, this systematic review aims to investigate the prevalence of frailty among patients with cancers during chemotherapy, explore risk factors associated with the development of frailty in this period, and guide early intervention.

## Methods

### Program Registration

This study’s protocol was prospectively registered with the PROSPERO (International Prospective Register of Systematic Reviews; CRD42024528132; dated April 3, 2024). The reporting process adhered to the PRISMA (Preferred Reporting Items for Systematic Reviews and Meta-Analyses) guidelines throughout the review development. Two deviations from the PROSPERO-registered protocol should be noted: (1) expanded synthesis framework: the prespecified quantitative meta-analysis was supplemented with a thematic qualitative analysis to enhance interpretation of frailty determinants, and (2) updated search timeframe: the literature search cutoff was extended from April 2024 to May 2025 to capture emerging evidence.

### Design

This systematic review was structured according to the PICo (Population, Interest, and Context) framework: Population (P): adult patients (≥18 years) diagnosed with malignant tumors through pathological examination, for whom chemotherapy constitutes a primary treatment modality; Interest (I): the occurrence of frailty and its predictive factors within this population; and Context (Co): clinical settings where chemotherapy is administered to patients with cancer.

### Search Methods

#### Search Strategy Development

The search strategy was developed through a systematic process involving multidisciplinary team discussions and a comprehensive review of previous literature on frailty assessment in oncology patients. Key elements of the strategy were informed by established frameworks in cancer-related frailty research, particularly those cited in recent Cochrane reviews and National Cancer Institute guidelines. Boolean operators and Medical Subject Headings terms were optimized through iterative testing across databases to ensure sensitivity and specificity. The search strategy was peer-reviewed by 2 evidence-based nursing experts (XD and JJ) with methodological expertise.

#### Database Search Methods

TW systematically searched the PubMed, Web of Science, Embase, China National Knowledge Infrastructure (CNKI), China Science and Technology Journal Database (VIP), and SinoMed from database inception through February 2025, with an updated search executed in May 2025 to capture newly published studies and incorporate critical evidence emerging during this review. The search strategy combined Medical Subject Headings, Emtree terms (for Embase), Chinese subject headings, and free-text keywords to identify studies on factors influencing frailty during chemotherapy in patients with cancer.

The keywords used were as follows: tumor, neoplasia*, cancer*, neoplasm, malignanc*, oncology, chemotherap*, chemoradiotherapy; asthenia, frail*, G-8, CGA, fried, VES-13, FI; cross-sectional stud*, cross-sectional survey*, quantitative research, investigat*, survey, influence factor, predictive factor, and predictor. The literature was searched according to the above standard retrieval methods and managed using the Note Express software to remove duplicate literature. Boolean operators (AND, OR, and NOT) and database-specific filters were applied (complete strategies available in Multimedia Appendix 1). Manual backward citation tracking of included papers and key reviews (eg, Cochrane reviews on cancer frailty) was performed to identify additional relevant studies. All retrieved records were imported into NoteExpress (version 3.7; Beijing Aegean Hailezhi Technology Co, LTD) for deduplication and management.

### Inclusion and Exclusion Criteria

Studies were selected according to the following criteria: (1) study design: observational studies (cohort, cross-sectional, or case-control studies) published in English or Chinese; (2) population: adults (≥18 years) with histologically confirmed malignant neoplasms at any stage and receiving or having received chemotherapeutic regimens; (3) outcome measures: quantified frailty status using validated instruments, including but not limited to the Fried frailty phenotype, Comprehensive Geriatric Assessment, Vulnerable Elder Survey-13 (VES-13), or Fatigue, Resistance, Ambulation, Illnesses, & Loss of Weight (FRAIL) scale [13]; and (4) data requirements: reporting multivariate-adjusted odds ratios with 95% CIs for frailty-associated factors or providing sufficient raw data for effect size calculation. Conversely, the exclusion criteria were (1) nonoriginal research (reviews or editorials), studies with ambiguous case definitions, or inaccessible full-text papers, and (2) publications with overlapping datasets without novel analyses.

### Quality Appraisal

Two independent reviewers (MZ and RZ) conducted the methodological evaluations of the included studies using design-specific tools. For cohort and case-control studies, the Newcastle-Ottawa Scale (NOS) [18] was applied to assess 3 critical domains: selection (representativeness of cohorts and exposure ascertainment), comparability (control for confounding variables), and outcome (validity of assessment methods and follow-up completeness). Studies were classified as low (0‐3 points), moderate (4-6), or high (7-9) quality [18]. Cross-sectional studies were evaluated using the Agency for Healthcare Research and Quality (AHRQ) checklist [19], which assigns scores (0‐11) across 11 criteria, including case definition clarity and consecutive participant enrollment. Each criterion was scored 1 (“yes”) or 0 (“no” or “unclear”), with final classifications as low (0-3), moderate (4-7), or high (8-11) quality [19]. Discrepancies in ratings were resolved through iterative team discussions, and unresolved cases were arbitrated by a third reviewer (XD), an evidence synthesis specialist.

### Data Extraction

Data extraction was conducted in a standardized double-blind manner by 2 investigators (TW and CY) who had completed evidence-based research training. The recorded variables encompassed bibliographic information (first author, publication year, and geographic region), study design characteristics (prospective or retrospective type, sample size, and tumor type), participant demographics (mean age with SD and diagnostic criteria), analytical parameters (frailty assessment tools and covariates adjusted in multivariate models), and outcome metrics represented by multivariate-adjusted odds ratios with 95% CIs. Frailty-associated factors were synthesized through cross-study comparative analysis, focusing on concordance patterns observed between independent odds ratio estimates.

### Statistical Analysis

Review Manager (version 5.4; Cochrane Collaboration) and R (version 4.4.3) were used to quantify associations between candidate variables and chemotherapy-induced frailty through the combined pooled odds ratios with 95% CIs. Heterogeneity was assessed through the Cochrane Q-test (significance threshold *P*<.10) and *I*^²^ statistics, with *I*^²^ ≥50% indicating substantial heterogeneity. Effect sizes were synthesized using fixed-effect models for homogeneous datasets (*I*^²^<50%) or random-effect models when significant heterogeneity existed. To investigate sources of heterogeneity, prespecified subgroup analyses were conducted by stratifying studies based on tumor type and frailty assessment tools. Sensitivity analyses were conducted by sequentially excluding studies with low quality ratings to assess the robustness of the pooled results. Publication bias was assessed using funnel plots for visual inspection when at least 10 studies were available. Publication bias was examined via Egger regression test, with *P*<.05 denoting potential bias. For predictors unsuitable for a meta-analysis (*I*^²^>50% or incomplete data reporting), a structured qualitative synthesis was performed. Factors reported in 2 or more studies were systematically categorized, with their associations classified by directionality (risk or protective) and strength of evidence.

## Results

### Search Outcomes

#### Initial Literature Search Results

In the initial search, 690 records were identified across 6 databases. After removing 448 duplicates and nonempirical studies using NoteExpress (version 3.7), 242 articles underwent title and abstract screening. Following the full-text assessment of 35 potentially eligible papers, 12 studies met the inclusion criteria.

#### Updated Search Rationale and Results

**Figure 1.**
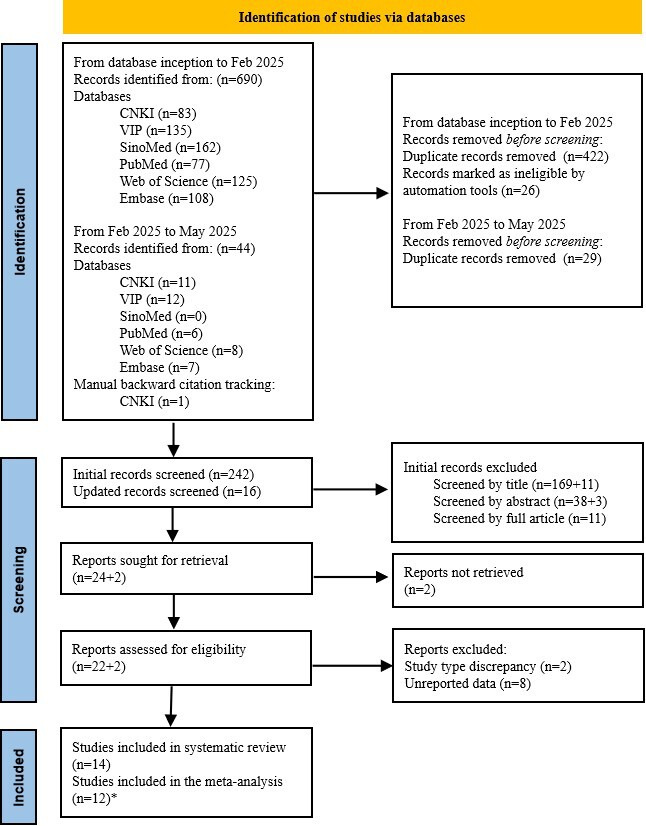
Literature screening flow chart. *There were 2 studies that were included in the systematic review but excluded from the meta-analysis due to use of quantitative frailty assessment scales and insufficient reporting of prevalence rates or odds ratio values.

To ensure comprehensiveness and capture recent publications, an updated search was conducted in May 2025. The search update was performed to include studies published between the initial search cutoff date and paper submission. The supplementary search yielded 44 records from the PubMed (n=6), Web of Science (n=8), Embase (n=7), CNKI (n=11), VIP (n=12), and SinoMed (n=0). Furthermore, manual backward citation tracking of the included studies and key reviews yielded 1 eligible article sourced from CNKI [20]. Following the removal of 29 duplicates and exclusion of 14 ineligible studies (11 excluded during title screening and 3 during abstract screening), 2 studies met the inclusion criteria and were included in the final analysis. The total number of included studies thus increased from 12 to 14 (Figure 1).

### Basic Features of the Included Studies

This systematic analysis incorporated 14 studies (8 cross-sectional [11,21-27], 3 cohort [28-30], 2 repeated cross-sectional [20,31], and 1 mixed-design [32]) involving 3879 patients with cancer. Most studies (9/14, 64.3%) were published between 2020 and 2025. Asian populations predominated (12/14, 85.7%), including 7 (50%) studies from mainland China, 2 (14.3%) from Taiwan, China, and 1 (7.1% each) each from Korea, Thailand, and Turkey. Two (14.3%) cohort studies were from North America.

Lung cancer was the most studied malignancy (5/14, 35.7%). Three cross-sectional studies (3/14, 21.4%) examined mixed malignancies, followed by single investigations of breast, pancreatic, colorectal, gastric, and biliary tract cancers. Sample sizes ranged from 75 to 1020 participants (median 185, IQR 124‐234). The weighted mean age across studies was 64.16 years (SE 9.07), with individual study means ranging from 53.4 to 71.4 years. Heterogeneous tools were used; Fried phenotype (4/14, 28.6%) and FRAIL scale variants (3/14, 21.4%) were the most common types. Frailty prevalence, reported in 11 (78.6%) studies, varied substantially by cancer type and assessment method: lowest in lung cancer cohorts using the Frailty Index based on Laboratory test data (4.9%) and highest in a mixed malignancy study involving the clinical frailty scale (67.3%).

Through the systematic screening of 14 studies, we identified 23 frailty-associated factors appearing in 2 or more studies. These were classified into 4 domains through independent dual-reviewer consensus (with third-party consultation resolving discrepancies). For demographic parameters, the most foundational domain included age, sex, education level, monthly family income, and marital status, with age being the most universally reported factor across studies. The socioeconomic-psychological factor domain integrated 3 psychosocial determinants, such as depression, anxiety, and social support, reflecting the interplay between mental health, resource accessibility, and care logistics. Clinical-treatment characteristics encompassed 13 factors directly related to disease progression and therapeutic interventions, including cancer type, cancer stage, transfer, chemotherapy frequency, surgical history, fall history, comorbidities, hypoproteinemia, Eastern Cooperative Oncology Group Performance Status (ECOG-PS), nutritional status, BMI, somatic symptom clusters, and emotional symptom clusters. Biological markers, comprising 2 objective laboratory parameters, highlighted physiological vulnerabilities through hemoglobin levels and white blood cell count. Notably, age (reported in 6 studies), depression (5 studies), and cancer stage (4 studies) emerged as the most frequently cited cross-cutting factors. The basic features of the included studies have been presented (Table 1).

**Table 1.** Basic characteristics of included studies (n=14).

Authors	Published time	Nationality	Research type	Neoplasms histologic type	Frailty assessment tool	Prevalence of frailty (%)	Influence factors^a^
Guolong et al [11]	2018	Guangzhou, China	Cross-sectional	Lung cancer	Comprehensive Frailty Assessment Instrument	—^b^	(1)(4)(8)(17)
Xia [21]	2020	Jiangsu, China	Cross-sectional	Malignant tumor	The Clinical Frailty Scale	67.3	(1)(2)(5)(7)(8)(9)(10)(12)(13)(14)(15)(17)(19) (20)(22)
Ho et al [22]	2021	Taiwan, China	Cross-sectional	Malignant tumor	Comprehensive Geriatric Assessment	58.1	(2)(5)(11)(21)
Boya [31]	2022	Henan, China	Repeated cross-sectional	Lung cancer	Chinese Version of Groningen Frailty Indicator	—	(2)(3)(6)(7)(9)(15)(16)(18)
Jeon et al [23]	2022	Korea	Cross-sectional	Stomach cancer, colorectal cancer, and lung cancer	The Korean Version of the FRAIL^c^ Scale	27.4	(8)
Xiaohuan and Zhijuan [24]	2023	Guangzhou, China	Cross-sectional	Lung cancer	The FRAIL Scale	45.4	(17)(18)
Gilmore et al [28]	2021	America	Cohort	Breast cancer	Modified Fried phenotype	—	(14)
Limpawattana et al [26]	2019	Thailand	Cross-sectional	Biliary tract cancer	The FRAIL Scale	12	(2)(6)
Duan et al [32]	2022	Taiwan, China	Mixed	Lung cancer	Fried Phenotype	23.2	(1)(2)(3)(4)(8)(9)(22)
Wang et al [29]	2019	Chengdu, China	Cohort	Lung cancer	FI-Lab^d^	4.9	(1)(10)(12)
Ngo-Huang et al [30]	2019	America	Cohort	Pancreatic cancer	Fried Phenotype	25.4	(11)(16)
Goktas et al [25]	2022	Turkey	Cross-sectional	Malignant tumor	Edmonton Frail Scale	51.6	(3)(5)(7)(9)(16)(21)(23)
Huijiao [20]	2023	Zhejiang, China	Repeated cross-sectional	Lung cancer	Chinese version of Groningen Frailty Indicator	38.4	(19)(20)
Jinhong et al [27]	2025	Guangzhou, China	Cross-sectional	Colorectal cancer	Fried Phenotype	65.86	(2)(8)(13)(14)(18)(19)(20)(23)

a (1) cancer stage, (2) age, (3) gender, (4) frequency of chemotherapy, (5) cancer type or disease diagnosis, (6) BMI, (7) education level, (8) depression, (9) monthly family income, (10) transfer, (11) Eastern Cooperative Oncology Group score (activity status), (12) surgery, (13) hemoglobin, (14) white blood cells, (15) social support, (16) comorbidity or chronic disease, (17) hypoproteinemia, (18) nutritional status, (19) somatic symptom cluster, (20) emotional symptom cluster, (21) marital status, (22) anxiety, and (23) history of falls.

b Not available.

c FRAIL: Fatigue, Resistance, Ambulation, Illnesses, & Loss of Weight.

d FI-Lab: Frailty Index based on Laboratory test data

### Quality Evaluation Results of the Included Studies

Methodological quality was rigorously evaluated using established tools: the 11-item AHRQ checklist for cross-sectional studies (Table 2) and the 8-item NOS for cohort studies (Table 3). The 11 cross-sectional studies achieved a mean AHRQ score of 8.8 (range: 7‐11), with 72.7% (8/11) scoring ≥8 points (high quality). Common methodological limitations included the inadequate description of missing data (7/11, 63.6%) and the absence of follow-up–related data (7/11, 63.6%). Three (27.3%) studies demonstrated a moderate risk of bias owing to the insufficient control of confounding variables. The 3 cohort studies attained high quality on NOS (mean score 8.0, range 7‐9) and demonstrated adequate outcome ascertainment and follow-up completeness. No studies were excluded based on quality thresholds, as per PRISMA guidelines for observational research synthesis.

**Table 2.** Quality evaluation results of cross-sectional studies for the 11-item AHRQ^a^ checklist (n=11).

Authors	①	②	③	④	⑤	⑥	⑦	⑧	⑨	⑩	⑪	AHRQ score	Quality level
Guolong et al, 2018 [11]	Y^b^	Y	Y	Y	Y	Y	Y	Y	N^c^	Y	N	9	High
Xia, 2020 [21]	Y	Y	Y	Y	Y	Y	Y	Y	N	Y	N	9	High
Ho et al, 2021 [22]	Y	Y	Y	N	Y	Y	N	N	N	Y	Y	7	Medium
Boya, 2022 [31]	Y	N	Y	Y	Y	Y	Y	Y	Y	Y	Y	10	High
Jeon et al, 2022 [23]	Y	Y	Y	Y	Y	Y	Y	Y	Y	Y	N	10	High
Xiaohuan and Zhijuan, 2023 [24]	Y	N	Y	Y	Y	Y	Y	Y	N	N	N	7	High
Limpawattana et al, 2019 [26]	Y	Y	Y	Y	Y	Y	N	Y	N	N	Y	8	Medium
Duan et al, 2022 [32]	Y	Y	Y	Y	Y	Y	Y	Y	Y	Y	N	10	High
Goktas et al, 2022 [25]	Y	Y	Y	Y	Y	Y	Y	N	N	Y	N	8	Medium
Huijiao, 2023 [20]	Y	Y	Y	Y	Y	Y	Y	Y	Y	Y	Y	11	High
Jinhong et al, 2025 [27]	Y	Y	Y	Y	N	Y	Y	Y	N	Y	N	8	High

a AHRQ: Agency for Healthcare Research and Quality.

b Y: yes.

c N: no.

**Table 3.** Quality evaluation results of the cohort studies for the 8-item NOS^a^ (n=3).

Authors	①	②	③	④	⑤	⑥	⑦	⑧	NOS score	Quality level
Wang et al, 2019 [29]	1	1	1	1	1	1	1	0	7	High
Ngo-Huang et al, 2019 [30]	1	1	1	2	1	1	1	0	8	High
Gilmore et al, 2021 [28]	1	1	1	2	1	1	1	1	9	High

a NOS: Newcastle-Ottawa Scale.

### Quantitative Analysis

#### Effect Size Pooled for Frailty Prevalence

**Figure 2.**
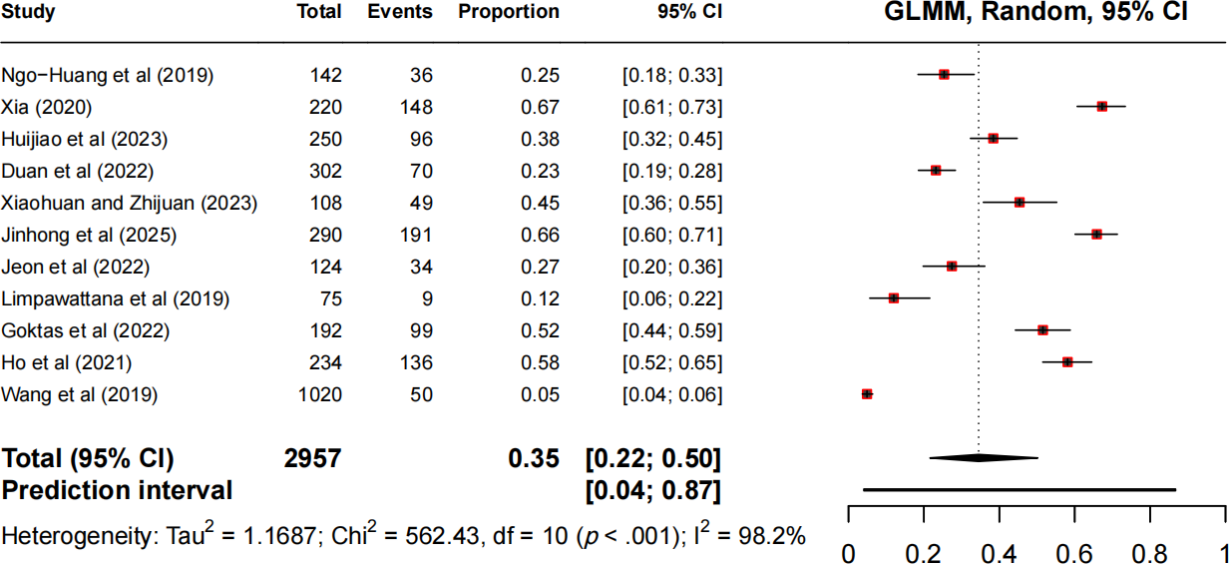
Forest chart of prevalence of frailty in patients with cancer during chemotherapy [20-27,29,30,32]. GLMM: generalized linear mixed model.

Guolong et al [11], Gilmore et al [28], and Boya [31] did not report the prevalence of frailty in patients with cancers. Therefore, data from 11 studies [20-27,29,30,32] encompassing 2957 patients with cancer undergoing chemotherapy were included in the meta-analysis. Among these patients, 918 were identified as frail, with individual study estimates ranging from 4.9% (95% CI 3.7%‐6.4%) to 67.3%. Notably, we observed an excessive between-study heterogeneity (*I*²=98.2%; *χ*²_10_=562.4, *P*<.001), suggesting that 98.2% of total variation originated from genuine differences rather than chance. Given this substantial heterogeneity, we used a random-effects model and interpreted pooled estimates with caution. The meta-analysis yielded a frailty prevalence of 34.5% (95% CI 21.6%‐50.2%), with prediction intervals ranging from 4% to 87% (Figure 2), suggesting that the true prevalence may differ substantially across clinical settings.

Subgroup analyses were conducted to explore potential sources of heterogeneity. Among cancer types, the lung cancer subgroup (7 studies, n=1804) exhibited a pooled frailty prevalence of 30% (95% CI 16%‐49%), with a profound between-study heterogeneity persisting (*I*^²^*=*97.5%) despite clinical homogeneity in cancer diagnosis. When stratified by assessment tools, studies involving the Fried Phenotype (3 studies, n=734) reported a prevalence of 37% (95% CI 18%‐61%) with substantial residual heterogeneity (*I*^²^=71.2%), whereas those involving the FRAIL scale (3 studies, n=307) showed a lower prevalence of 27% (95% CI 14%‐45%) and moderate heterogeneity (*I*^²^=46.8%). Although subgrouping by assessment tool reduced heterogeneity for the FRAIL scale, all subgroup estimates retained considerable variability (*I*^²^>45%), suggesting that unmeasured factors contributed to the observed differences. These results suggest that the cancer type and assessment method do not fully explain the excessive heterogeneity in frailty prevalence among chemotherapy patients.

#### Combination of the Effect Sizes of Frailty Predictors

Influencing factors reported in 2 or more studies (with available effect estimates) were included in the meta-analysis. Using a fixed-effects model for meta-analysis, the results indicated that the heterogeneity values for cancer stage (*I*^²^=14%), chemotherapy frequency (*I*^²^=46%), transfer (*I*^²^=36%), hemoglobin (*I*^²^=0%), white blood cell (*I*²=0%), comorbidity (*I*^²^=12%), and hypoproteinemia (*I*^²^=0%) were within acceptable limits. The 7 factors had statistically significant effects on frailty among patients with cancer during chemotherapy (*P*<.05, Table 4).

**Table 4.** Meta-analysis results of frailty predictors among cancer patients undergoing chemotherapy.

Influencing factors	Number of studies	Heterogeneity		Model	Meta-analysis results	
		*I*² (%)	*P* value		Odds ratio (95% CI)	*P* value
Cancer stage	4	14	.32	FE^a^	1.99 (1.64‐2.42)	<.001
Age	5	86	<.001	RE^b^	1.75 (1.24‐2.47)	.002
Gender	2	84	.01	RE	0.64 (0.24‐1.74)	.38
Frequency of chemotherapy	2	46	.17	FE	2.60 (1.83‐3.70)	<.001
Education level	2	62	.11	RE	0.48 (0.31‐0.74)	.001
Depression	4	84	<.001	RE	1.63 (1.19‐2.23)	.002
Monthly family income	2	85	.009	RE	0.84 (0.38‐1.86)	.66
Transfer	2	36	.21	FE	2.18 (1.50‐3.17)	<.001
ECOG-PS^c^	2	70	.07	RE	3.73 (1.83‐7.60)	<.001
Hemoglobin	2	0	.55	FE	0.29 (0.18‐0.47)	<.001
White blood cells	2	0	.81	FE	0.37 (0.21‐0.65)	<.001
Comorbidity or chronic disease	2	12	.29	FE	1.93 (1.30‐2.86)	.001
Hypoproteinemia	2	0	.34	FE	1.74 (1.31‐2.30)	<.001
Nutritional status	2	59	.12	RE	1.51 (0.92‐2.48)	.10
Somatic symptom cluster	3	98	<.001	RE	1.42 (1.09‐1.86)	.01
Emotional symptom cluster	3	93	<.001	RE	1.27 (1.04‐1.55)	.02

a FE: fixed effects.

b RE: random effects.

c ECOG-PS: Eastern Cooperative Oncology Group Performance Status.

### Sensitivity Analysis

We performed sensitivity analyses using a two-step approach: (1) the sequential exclusion of individual studies when subgroup analyses included 3 or more studies, and (2) the targeted removal of studies contributing to substantial heterogeneity (*I*^²^>50%). In assessing the robustness of our findings, sensitivity analyses revealed critical insights into heterogeneity patterns (Multimedia Appendix 2). The exclusion of the study by Wang et al [29], which reported the lowest frailty prevalence (4.9%, 95% CI 3.7%‐6.4%), preserved excessive residual heterogeneity (*I*^²^=96%, *P*<.001), indicating alternative variability sources such as methodological discrepancies in frailty assessment tools (5 distinct instruments used), clinical diversity across cancer types, and geocultural variations in frailty conceptualization contrasting Asian and Western cohorts.

Subsequent domain-specific analyses demonstrated differential heterogeneity profiles. The removal of the study by Limpawattana et al [26] from age-related factor analyses (5 studies) substantially reduced heterogeneity to moderate thresholds (*I*^²^=44%, *P*=.15) while preserving effect stability (pre-exclusion odds ratio 1.75, 95% CI 1.24‐2.47 vs postexclusion odds ratio 1.95, 95% CI 1.52‐2.49). For depressive symptom studies (4 articles), sequential exclusion procedures yielded minimal heterogeneity fluctuations (Δ*I*^²^<8%) with consistent effect magnitudes. Physical symptom cluster analyses (3 studies) maintained stable effect estimates despite persistent high heterogeneity (*I*^²^>65%). Notably, the exclusion of the study by Xia [21] in emotional symptom cluster investigations (3 studies) effectively resolved heterogeneity (*I*^²^=21%, *P*=.26) without directional change in effects (pre-exclusion odds ratio 1.27, 95% CI 1.04‐1.55 vs postexclusion odds ratio 1.37, 95% CI 1.20‐1.57).

### Publication Bias Analysis

**Figure 3.**
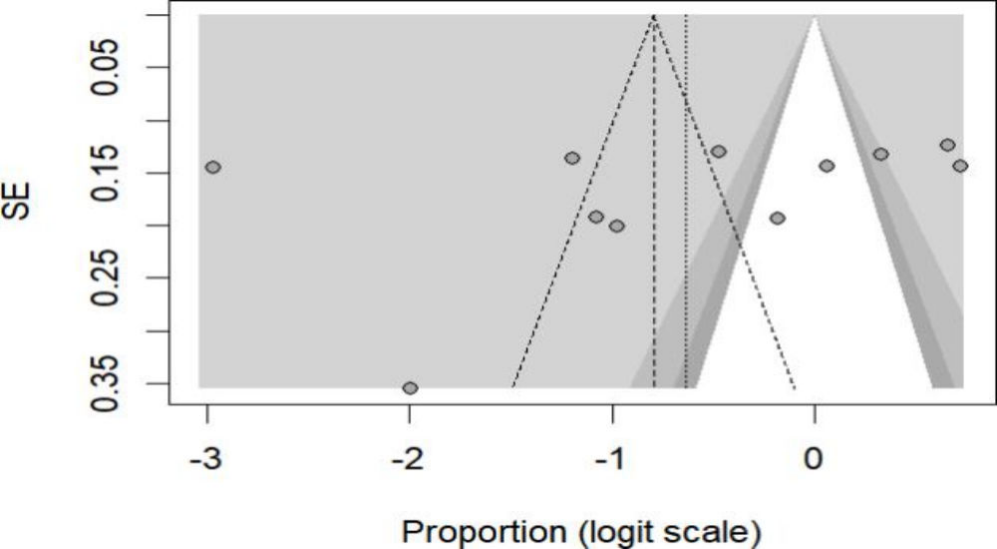
Funnel plot of the prevalence of frailty.

Potential publication bias was evaluated using funnel plots for outcomes with 10 or more studies. In the meta-analysis of frailty prevalence, which included 11 studies (Figure 3), no significant publication bias was detected using the Egger test (*t*_9_=−0.96,, *P*=.36). However, the presence of substantial heterogeneity necessitates a cautious interpretation of the pooled results.

Owing to the few studies included in the meta-analysis (applied to outcomes with 3 or more studies, including cancer stage, age, and depression), Egger test was used to assess potential publication bias. While no significant publication bias was detected for the cancer stage (*P*>.05), statistically significant asymmetry was observed in the analyses of age (*P*=.04) and depression (*P*=.02), suggesting publication bias. Notably, the Egger test has reduced statistical power when fewer than 10 studies are included and may overestimate effect sizes or be confounded by heterogeneity. Therefore, these results were interpreted with caution, supplemented by the visual inspection of funnel plots and comprehensive sensitivity analyses.

### Qualitative Analysis

Our systematic review revealed 7 factors associated with frailty in chemotherapy-treated patients with cancers who were consistently reported in at least 2 studies: cancer type, surgical history, BMI, social support, marital status, anxiety, and history of falls. However, quantitative synthesis through odds ratio pooling was precluded owing to insufficient data reporting across these studies. For 9 additional factors demonstrating substantial heterogeneity (*I*²>50%), a descriptive synthesis was conducted.

Sociodemographic factors included (1) advanced age: 5 studies [21,22,26,27,32] consistently associated old age with increased frailty risk; (2) female sex: 2 studies [25,32] reported higher frailty prevalence in females than in males, potentially linked to lean mass reduction and postmenopausal endocrine shifts; (3) marital status: 2 studies [11,32] suggested unmarried status as a risk factor; however, the effect estimates were inconsistent; (4) education level: low education was associated with frailty in 2 studies [21,25]; and (5) monthly household income: 2 studies [21,25] indicated low income as a risk factor; however, the pooled results were nonsignificant.

Disease or treatment-related factors included (1) cancer type: frailty prevalence varied markedly by malignancy: 37.2% in breast cancer [28], 45.4% in lung cancer [24], and 25.4% in pancreatic cancer [30]; (2) surgical history: 2 studies [21,29] linked prior surgery to frailty progression; however, the mechanisms remain unclear; and (3) history of falls: 2 studies [25,27] implicated falls as a precursor and consequence of frailty.

Psychosocial and socioeconomic factors included (1) social support: 2 studies [21,31] highlighted inadequate social support as a contributor to frailty; (2) depression: 5 studies [11,21,23,27,32] indicated that patients with high depression severity exhibited an increased susceptibility to frailty, demonstrating a bidirectional association between frailty and depression; (3) anxiety: 2 studies [21,32] associated anxiety with frailty severity, particularly in advanced disease stages; and (4) emotional symptom cluster: 3 studies [20,21,27] demonstrated emotional distress as a frailty accelerator.

Symptom-functional domains included (1) ECOG-PS: 2 studies [22,30] reported that patients with ECOG-PS scores ≥2 exhibited heightened susceptibility to frailty, demonstrating that impaired mobility contributes to frailty progression through deconditioning cycles; (2) nutritional status: 3 investigations [24,27,31] identified that individuals at risk of malnutrition had a high likelihood of developing frailty; (3) BMI: 2 studies [26,31] recognized abnormal BMI values (underweight and overweight statuses) as independent risk factors for frailty; however, one study failed to report the data on the significant association between underweight or overweight status and the progression of frailty; and (4) somatic symptom cluster: 3 studies [20,21,27] indicated that multisystem symptom burden is a critical correlate of frailty.

### Heterogeneity and Evidence Strength

High-confidence evidence (3 or more consistent studies) supported age, depression, nutritional status, emotional symptom cluster, and somatic symptom cluster as frailty predictors. Other factors, such as marital status and anxiety, required further investigation owing to limited or conflicting data.

## Discussion

### Principal Results

This meta-analysis revealed a 35% (95% CI 22%‐50%) pooled prevalence of frailty among patients with cancer undergoing chemotherapy. Notably, substantial heterogeneity was observed (*I*^²^=98.2%, *P*<.001) in frailty prevalence among chemotherapy patients, which persisted even after subgrouping by cancer type and assessment tools. Fixed-effects modeling identified 7 robust clinical predictors: advanced cancer stage, frequent chemotherapy cycles, care transitions, anemia, white blood cell count, comorbidity burden, and hypoproteinemia, all demonstrating low heterogeneity (*I*^²^=0%‐46%). Qualitative synthesis demonstrated strong evidentiary support for age, depression, nutritional status, emotional symptom clusters, and somatic symptom clusters as frailty determinants, with multiple studies consistently reinforcing these associations across heterogeneous populations. Notably, while quantitative synthesis confirmed biological and treatment-related predictors, qualitative findings highlighted understudied socioeconomic (marital status, education, and income) and symptom cluster determinants. The persistent residual heterogeneity suggests unmeasured contextual factors, potentially including treatment intensity variations and health care access disparities, may mediate frailty trajectories in this population.

### Limitations

This study has several limitations that should be acknowledged. First, regarding the included studies, (1) the majority were observational investigations with small sample sizes, and multicenter large-scale cohort studies remain scarce; and (2) significant methodological diversity existed across studies, including heterogeneous case sources, inconsistent application of frailty assessment tools, and the absence of standardized diagnostic criteria for frailty, which introduced clinical and statistical heterogeneity.

Second, limitations inherent to our systematic review methods include: (1) language restrictions in our search strategy, which excluded studies not published in Chinese or English, may have omitted relevant evidence from regions where these languages are not primary; (2) there are inherent constraints in the use of Egger test to detect publication bias with limited study numbers (n<10), where false-positive rates increase significantly; (3) despite comprehensive database searches, the possible omission of gray literature (eg, conference abstracts and unpublished datasets) might have skewed effect estimates toward statistically significant findings; and (4) our meta-analytic approach could not fully disentangle confounding factors due to insufficient primary data, limiting causal inference.

These constraints highlight the need for future research using standardized frailty criteria, prospective multinational cohorts, and individual-patient-data meta-analyses to address these gaps.

### Comparison With Prior Work

The pooled prevalence of frailty observed in chemotherapy-treated patients with cancers exceeded the 34% prevalence reported in general cancer populations [7] and the 14.2% prevalence documented in noncancer patients admitted to intensive care after surgery [33]. Systematic evidence synthesis revealed substantial heterogeneity in frailty prevalence among older patients with cancer, with a review of 20 studies (n=2916) reporting a median prevalence of 43% (range 4%‐86%) [34]. This discrepancy may reflect our study’s inclusion of chemotherapy patients without age restrictions. This accelerated frailty progression aligns with evidence showing the heightened vulnerability of older patients with cancers to adverse health outcomes compared with that of younger counterparts [35]. Measurement variability across patient populations and assessment tools increased this heterogeneity [35].

Notably, longitudinal data from Gilmore et al [36] demonstrated the profound impact of chemotherapy on frailty trajectories in patients with breast cancer aged ≥50 years. Using modified Fried criteria, the proportion of participants meeting frailty thresholds (Fried score ≥3) increased from 13% prechemotherapy to 46% posttreatment. The pathophysiology of chemotherapy-associated frailty involves complex interactions between aging mechanisms and cytotoxic stress. Cellular senescence and stem cell depletion during typical aging processes reduce physiological reserve capacity [1,35], whereas chemotherapy-induced damage exacerbates these deficits through impaired tissue repair mechanisms [37,38]. This dual burden manifests clinically as accelerated functional decline, weight loss, fatigue, and impaired activities of daily living during treatment [28].

From a clinical outcome perspective, frailty status is an independent predictor for multiple adverse treatment effects. It is associated with reduced chemotherapy response rates [10] and increased risk of treatment failure even after successful tumor burden reduction [9]. Furthermore, frail patients exhibit elevated mortality risks following surgical interventions and chemotherapy regimens [7,29,39]. Notably, frailty correlates with multidimensional quality of life impairments, manifesting as physical functional decline, psychological distress, and social participation limitations [7,12]. These interconnected outcomes underscore the role of frailty as a critical prognostic indicator across the cancer care continuum.

Moreover, the dynamic nature of frailty permits early intervention. Timely identification through validated assessment tools enables the implementation of targeted prehabilitation programs. Current evidence suggests that these interventions prevent, reverse, or delay frailty progression, reducing adverse outcomes and the associated socioeconomic burdens in populations with cancers [7]. This synthesis highlights the critical need for standardized frailty assessments in chemotherapy decision-making and underscores the modifiable nature of frailty when detected in preclinical stages.

Our findings align with Fried frailty phenotype [3] and the biopsychosocial model of frailty and emphasize frailty as a multidimensional syndrome requiring integrated assessment frameworks arising from interconnected biological, clinical, and socioeconomic mechanisms. The synergy between tumor biology (eg, metastatic inflammation) and treatment toxicity (eg, platinum-induced myelosuppression) creates a “double-hit” mechanism in older patients, where chemotherapy amplifies mitochondrial dysfunction and epigenetic aging, aligning with models of accelerated aging in cancer survivors. This biological-clinical interplay is compounded by socioeconomic mediation of risk. Severe depression elevates frailty risk by 10.7-fold through neuroendocrine-immune crosstalk; however, intervention effectiveness may be limited by disparities in care access, underscoring the need for policy reforms to address therapeutic inequities. Similarly, functional-nutritional vicious cycles driven by Eastern Cooperative Oncology Group ≥2 and hypoalbuminemia perpetuate frailty progression—mobility restriction exacerbates sarcopenia, while protein catabolism impairs recovery, highlighting the urgency for prehabilitation protocols integrating physical therapy and nutritional optimization. These findings highlight the significance of stage-adapted frailty screening tools that incorporate biomarkers (albumin or hemoglobin) and psychosocial profiles in clinical settings. For high-risk patients, comorbidity-centered dose modifications and early palliative care referrals may mitigate compounding morbidity, bridging the gap between mechanistic understanding and personalized geriatric oncology care.

With the aging global population, cancer and frailty have become critical health challenges for older adults. Frailty in older patients with cancer is associated with numerous adverse clinical outcomes, including functional decline, falls, impaired mobility, disability, increased hospitalization rates, and mortality [22]. This structured taxonomy enables clinicians to prioritize multidimensional assessments while guiding researchers in developing targeted frailty intervention frameworks. Early identification of individuals at high frailty risk, particularly through age-based screening, and the implementation of tailored interventions are crucial for reducing adverse outcomes. The interaction between biological markers and psychosocial factors particularly warrants further investigation, given their co-occurrence in multiple studies.

Prevention and management strategies for frailty during chemotherapy should also consider individual sex differences, socioeconomic status, and overall health. The disparity in frailty prevalence observed among patients with different malignancies during chemotherapy possibly reflects underlying tumor-specific pathophysiological mechanisms and regimen-related toxicities. For example, the elevated frailty burden in lung cancer may correlate with frequent late-stage diagnoses and high chemotherapy-induced immunosuppression, whereas metabolic derangements and rapid functional decline in pancreatic cancer may accelerate frailty trajectories. Such cancer-specific patterns emphasize the need for tailored screening protocols aligned with tumor biology and treatment regimens.

### Conclusions

This systematic review and meta-analysis revealed a substantial prevalence of frailty among chemotherapy-treated populations with cancers. Therefore, we suggest the systematic implementation of validated frailty screening protocols in routine clinical assessments before chemotherapy initiation. Preliminary evidence of frailty risk factors for patients with cancer during the chemotherapy stage was obtained through systematic evaluation. Based on these risk factors, early prevention and management strategies should be implemented to mitigate the impacts of frailty on chemotherapy and improve treatment outcomes. Future in-depth multicenter, large-sample studies are required to explore the role of depression in frailty during chemotherapy and comprehensively evaluate the various factors influencing frailty in patients with cancers undergoing chemotherapy, sufficiently guiding clinical practice.

## Supplementary material

10.2196/69936 Multimedia Appendix 1 Searching strategies (from database inception to February 2025).

10.2196/69936 Multimedia Appendix 2 Sensitivity analysis data.

10.2196/69936 Checklist 1 PRISMA 2020 checklist. PRISMA: Preferred Reporting Items for Systematic Reviews and Meta-Analyses.

## Abbreviations

AHRQ: Agency for Healthcare Research and Quality
CNKI: China National Knowledge Infrastructure
ECOG-PS: Eastern Cooperative Oncology Group performance status
FRAIL: Fatigue, Resistance, Ambulation, Illnesses, & Loss of Weight
NOS: Newcastle-Ottawa Scale
PICo: Population, Interest, and Context
PRISMA: Preferred Reporting Items for Systematic Reviews and Meta-Analyses
PROSPERO: International Prospective Register of Systematic Reviews
VES-13: Vulnerable Elder Survey-13
