# Supplementary material for: Prevalence of Frailty and Its Predictors Among Patients With Cancer at the Chemotherapy Stage: Systematic Review
Source: JMIR Cancer. 2025 Jul 24;11:e69936. doi: 10.2196/69936 (PMC12289293; doi:10.2196/69936)
Supplement: Multimedia Appendix 1 [file cancer-v11-e69936-s001.docx]

| **Items** | **Searching Strategy** | **Results** |
| --- | --- | --- |
| **CNKI** | | |
| #1 | （篇名：衰弱）AND（篇名：癌症 + 癌 + 肿瘤 + 化疗 + 放化疗） | 83 |
| **VIP** | | |
| #1 | 篇名=衰弱 AND 篇名=癌症 or 癌 or 肿瘤 or 化疗 or 放化疗 | 135 |
| **Sinomed** | | |
| #1 | ( "癌症"[中文标题] OR "癌"[中文标题] OR "肿瘤"[中文标题] OR "化疗"[中文标题] OR "放化疗"[中文标题]) AND( "衰弱"[中文标题]) | 162 |
| **Pubmed** | | |
| #1 | "tumor"[Title] OR "neoplasia*"[Title] OR "cancer*"[Title] OR "neoplasm*"[Title] OR "malignan*"[Title] OR "oncology"[Title] OR "chemotherap*"[Title] OR "chemoradiotherap*"[Title] | 2,029,972 |
| #2 | "asthenia"[Title] OR "frail*"[Title] OR "G-8"[Title] OR "CGA"[Title] OR "fried"[Title] OR "VES-13"[Title] OR "FI"[Title] | 21,893 |
| #3 | "cross-sectional"[Title] OR "cross-sectional"[Title] OR "quantitative research"[Title] OR "investigat*"[Title] OR "survey*"[Title] OR "influence factor"[Title] OR "predictive factor"[Title] OR "predictor*"[Title] | 622,625 |
| #4 | #1 and #2 and #3 | 77 |
| **Web of Science** | | |
| #1 | TI=(tumor OR neoplasia* OR cancer* OR neoplasm OR malignan* OR oncology OR chemotherap* OR chemoradiotherap*) | 3,654,704 |
| #2 | TI=(asthenia OR frail* OR G-8 OR CGA OR fried OR VES-13 OR FI) | 58553 |
| #3 | TI=(cross-sectional OR “cross sectional” OR “quantitative research” OR investigat* OR survey* OR “influence factor” OR “predictive factor” OR predictor) | 1,884,597 |
| #4 | #1 and #2 and #3 | 128 |
| #5 | #4 and English or Chinese (Languages) | 125 |
| **Embase** | | |
| #1 | tumor:ti OR neoplasia*:ti OR cancer:ti OR neoplasm:ti OR malignant*:ti OR oncology:ti OR chemotherap*:ti OR chemoradiotherap*:ti | 2632361 |
| #2 | asthenia:ti OR frail*:ti OR 'g-8':ti OR cga:ti OR fried:ti OR 'ves 13':ti OR fi:ti | 30566 |
| #3 | 'cross sectional':ti OR 'quantitative research':ti OR investigat*:ti OR survey*:ti OR 'influence factor':ti OR 'predictive factor':ti OR predictor*:ti | 800504 |
| #4 | #1 and #2 and #3 | 109 |
| #5 | #4 AND ([chinese]/lim OR [english]/lim) | 108 |
