# Supplementary material for: Prevalence of Frailty and Its Predictors Among Patients With Cancer at the Chemotherapy Stage: Systematic Review
Source: JMIR Cancer. 2025 Jul 24;11:e69936. doi: 10.2196/69936 (PMC12289293; doi:10.2196/69936)
Supplement: Multimedia Appendix 2 [file cancer-v11-e69936-s002.docx]

**Sensitivity Analysis**

We performed sensitivity analyses using a two-step approach: (1) the sequential exclusion of individual studies when subgroup analyses included three or more studies, and (2) the targeted removal of studies contributing to substantial heterogeneity.

1. **Sensitivity Analysis of Frailty Prevalence Estimates**

A leave-one-out sensitivity analysis was sequentially performed by excluding each of the 11 studies included in the meta-analysis to evaluate the influence of individual studies on the pooled effect estimates. Figure S1 presents the summary effect sizes (proportions), 95% confidence intervals (95% CI), heterogeneity statistics (Tau², Tau, I²), and related statistical test results after each study removal.


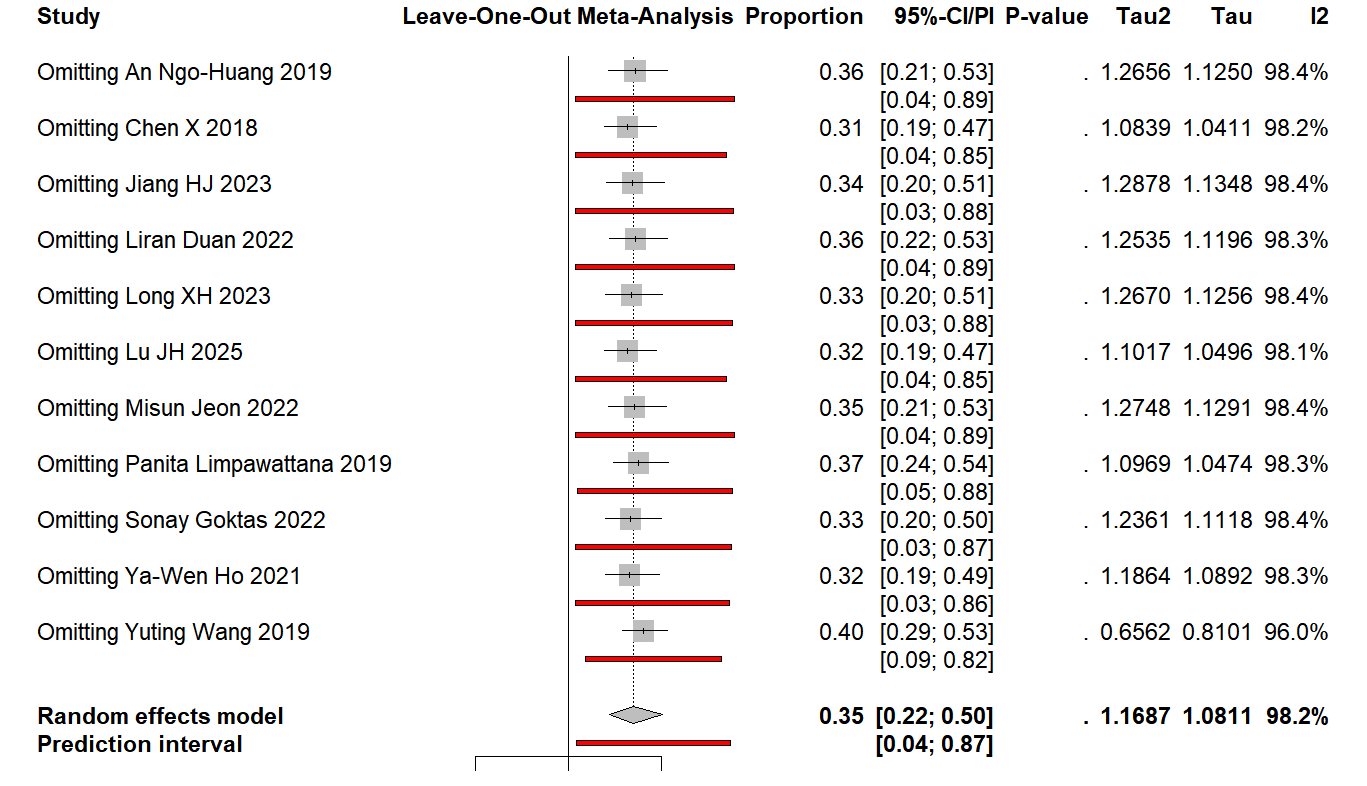


**Figure S1. Sensitivity analysis of frailty prevalence estimates.**

The sequential exclusion analysis revealed that the pooled prevalence estimates ranged from 0.31 to 0.40 across individual study removals, consistently fluctuating around the original pooled estimate (0.35, 95% CI [0.22; 0.50]). All recalculated confidence intervals remained wide and encompassed or approached the primary effect estimate, indicating limited influence of individual study removal on the overall effect estimates. These findings suggest robust meta-analytic results.

Notably, the highest prevalence estimate (0.40) was observed after excluding the study by Yuting Wang et al. (2019), implying a potential downward contribution of this study to the original pooled effect. Conversely, the lowest estimate (0.31) emerged following the exclusion of Chen X et al. (2018). However, neither exclusion substantially altered the overall interpretation of the results.

Heterogeneity metrics remained persistently high across all sensitivity analyses, with I² values ranging from 96.0% to 98.4%, confirming substantial between-study heterogeneity. Both Tau² and Tau values exhibited minimal fluctuations, and the dispersion estimates remained stable regardless of study exclusion, suggesting that the observed heterogeneity was inherent to the overall dataset rather than driven by any single study.

To sum up, the sensitivity analysis demonstrates the robustness of the meta-analytic results, as neither the pooled prevalence estimates nor heterogeneity patterns were meaningfully affected by the exclusion of individual studies. However, the persistently high heterogeneity and wide prediction intervals highlight substantial variability across studies.

1. **Age**

As shown in Figure S2, the pooled odds ratio remained within the range of 1.55 to 1.95 after sequential exclusion of individual studies. All confidence intervals did not cross the null value of 1, indicating that the results retained statistical significance and the overall effect direction remained consistent throughout sensitivity analyses. The maximum odds ratio (OR=1.95, 95% CI [1.52; 2.49]) was observed when excluding the study by Panita Limpawattana (2019), suggesting this particular study might have contributed to attenuating the overall effect size. Conversely, the lowest odds ratio (OR=1.55, 95% CI [1.13; 2.12]) emerged after removing the study by Lu JH (2025), implying this research potentially exerted an upward influence on the overall effect estimate.


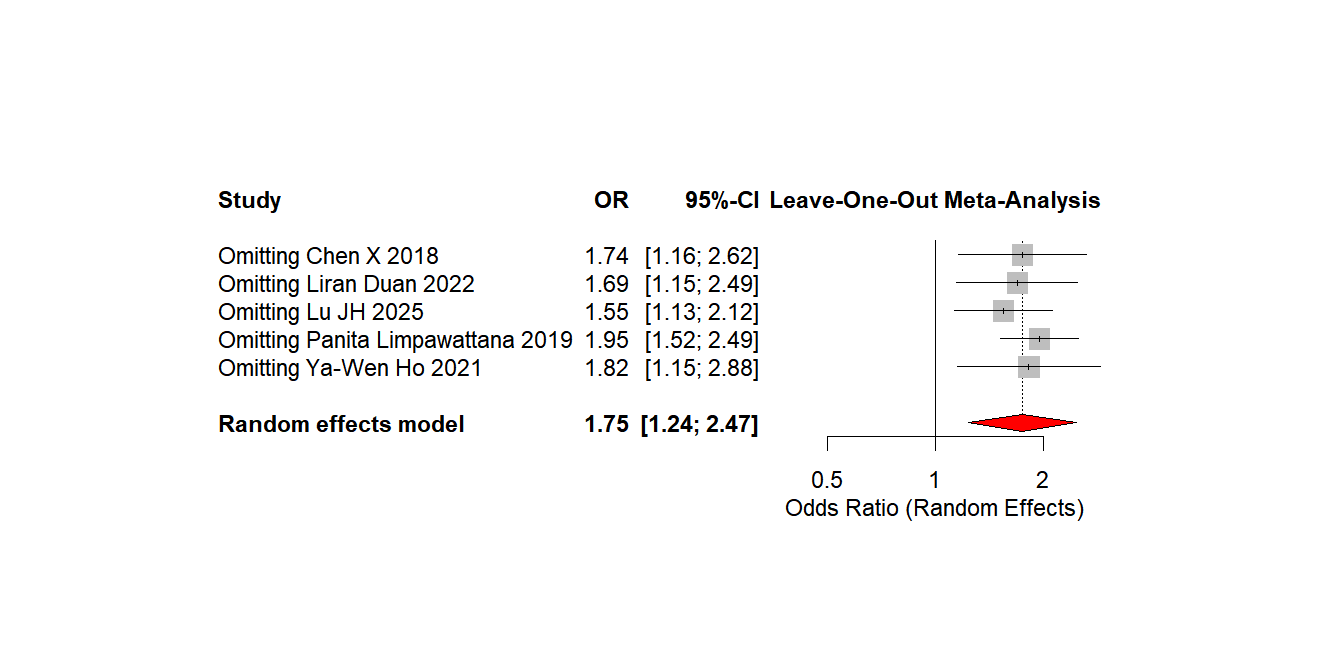


**Figure S2. Sensitivity analysis of the association between age and frailty.**

1. **Depression**

As depicted in Figure S3, we systematically excluded each of the four incorporated studies from the meta-analysis and recalculated the pooled OR with corresponding 95% CI. The sensitivity analysis revealed that the adjusted ORs fluctuated between 1.44 and 1.91 across all exclusion scenarios. Notably, all recalculated confidence intervals remained statistically significant (none crossing the null value of 1). The maximum effect estimate (OR=1.91, 95% CI [1.24; 2.94]) emerged after excluding the study by Chen X (2018), implying this investigation may have partially attenuated the overall effect magnitude. Conversely, the lowest pooled OR (1.44, 95% CI [1.09; 1.89]) was observed upon removal of Zhang GL's study (2018), suggesting this particular research might have contributed to an upward bias in the primary analysis. These findings collectively demonstrate the robustness of the pooled estimate, as neither exclusion of individual studies nor directional variations in effect sizes substantially altered the statistical significance or magnitude of the observed association.


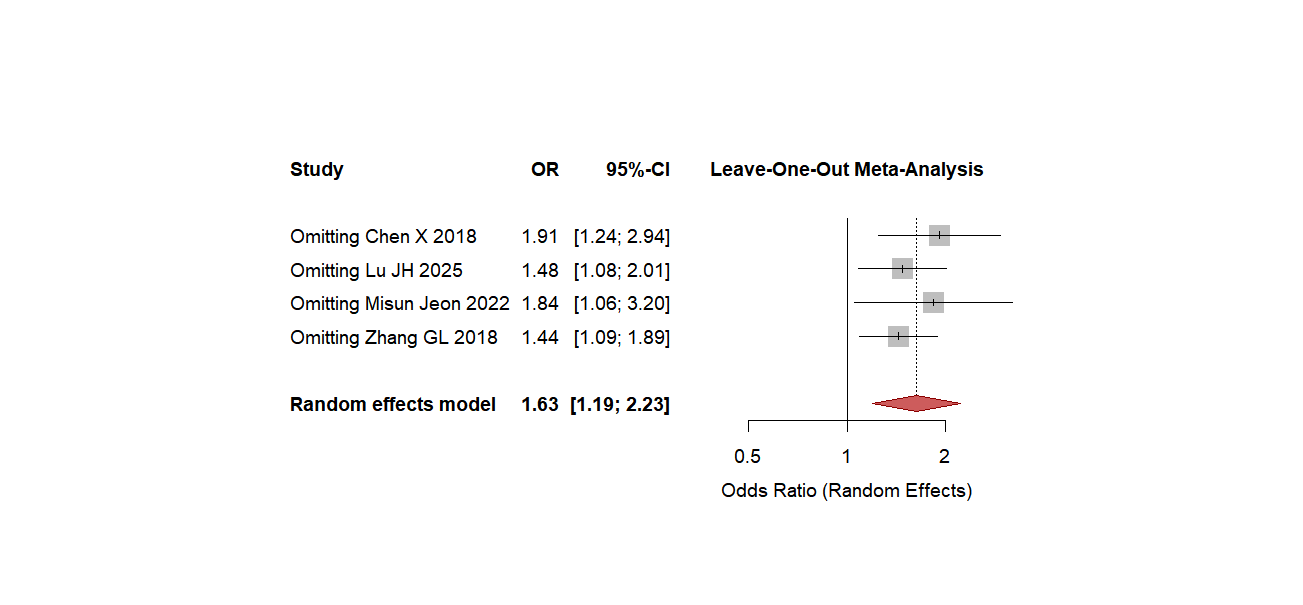


**Figure S3. Sensitivity analysis of the association between depression and frailty.**

1. **Somatic symptom cluster**

As illustrated in Figure S4, sensitivity analysis was conducted by sequentially excluding three pivotal studies to assess their influence on the pooled OR and 95% CI. Exclusion of Lu JH (2025) reduced the OR to 1.27 (95% CI [0.94, 1.73]), with the confidence interval crossing the null value of 1, thereby abolishing statistical significance. This indicates that Lu JH’s study critically contributed to the overall effect estimate, as its removal destabilized the significance of the meta-analytic result. Subsequent exclusion of Jiang HJ (2023) yielded an OR of 1.46 (95% CI [0.79, 2.70]), characterized by a widened confidence interval spanning the null value, which reflects substantial variability and potential instability in either sample characteristics or methodological rigor. Conversely, removing Chen X (2018) strengthened the association (OR=1.66, 95% CI [1.23, 2.24]), with statistical significance preserved, suggesting that this study modestly attenuated the original pooled estimate, possibly due to conservative bias. The meta-analytic conclusions demonstrated marked sensitivity to the exclusion of Lu JH (2025) and Jiang HJ (2023), as their removal nullified statistical significance, highlighting their disproportionate weight in driving the overall effect. Notably, the pronounced expansion of confidence intervals, particularly after excluding Jiang HJ (2023), signals underlying clinical or methodological heterogeneity across studies, which compromises the stability of the findings. While the direction of association remained consistent in most scenarios, the fragility of significance thresholds upon specific exclusions underscores the need for cautious interpretation of the pooled effect size in the context of potential heterogeneity.


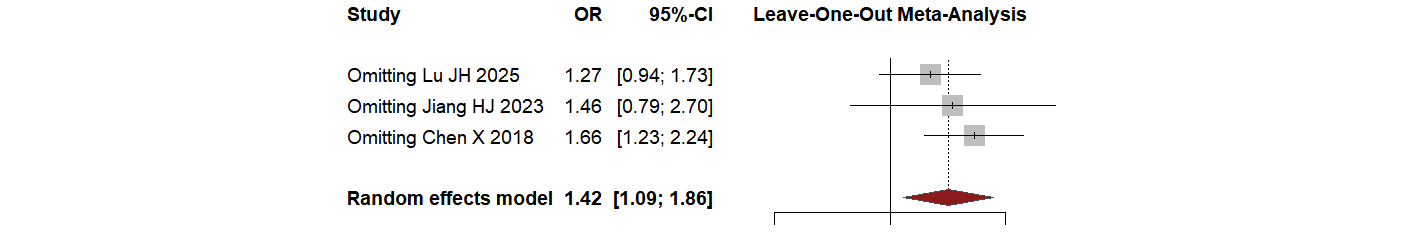


**Figure S4. Sensitivity Analysis of Somatic Symptom Cluster.**

1. **Emotional symptom cluster**

A sensitivity analysis was performed by sequentially excluding individual studies to assess their impact on the pooled odds ratio (OR) and 95% confidence interval (95% CI). Exclusion of Chen X (2018) resulted in an OR of 1.37 (95% CI [1.20, 1.57]), with statistical significance preserved and the CI excluding the null value (1), indicating that while this study contributed to the overall effect, its removal did not undermine the robustness of the meta-analytic conclusion. In contrast, removing Jiang HJ (2023) reduced the OR to 1.28 (95% CI [0.85, 1.93]), with the CI spanning the null value and statistical significance lost, suggesting this study plays a pivotal role in maintaining the significance of the pooled effect. Similarly, exclusion of Lu JH (2025) yielded an OR of 1.20 (95% CI [0.96, 1.49]), where the CI also encompassed 1, further demonstrating its substantial contribution to the overall effect estimate. These findings collectively indicate that while the meta-analysis demonstrates statistically significant pooled effects, the results are highly dependent on specific studies. The removal of Jiang HJ (2023) or Lu JH (2025) destabilized the significance thresholds, revealing limited stability and potential overreliance on individual datasets. The sensitivity analysis underscores the need for cautious interpretation of the meta-analytic conclusions, as the observed associations may be vulnerable to methodological heterogeneity or sampling variability inherent in the included studies.


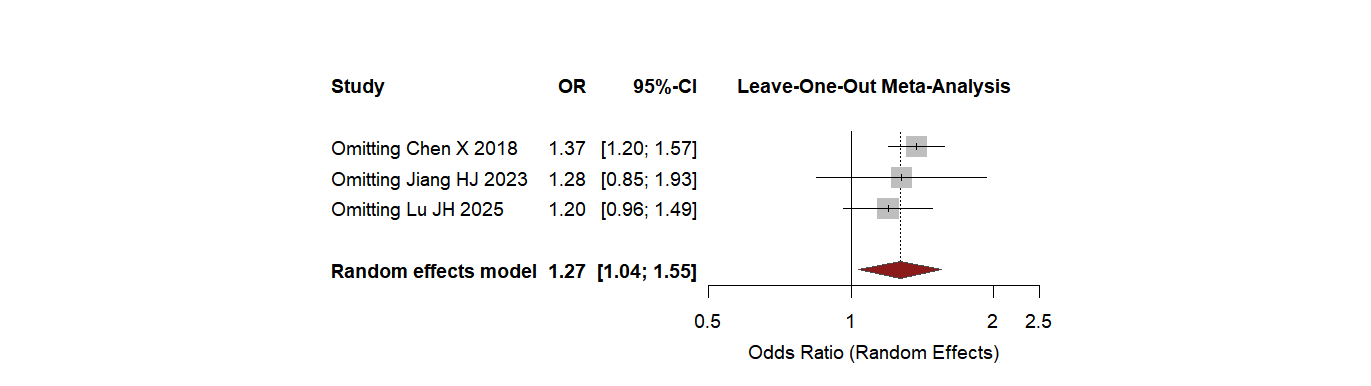


**Figure S5. Sensitivity Analysis of Emotional symptom cluster.**
